# Associations Between Nicotine Dependence and Health-Promoting Lifestyle Behaviors Among Nursing Students

**DOI:** 10.1192/j.eurpsy.2026.10870

**Published:** 2026-07-31

**Authors:** A. Kchaou, A. Triki, N. Kotti, F. Dhouib, W. Ben Messaoud, M. Hajjaji, K. J. Hammami

**Affiliations:** 1 Occupational Health, Hedi Chaker University Hospital; 2 Higher Institute of Nursing Sciences of Sfax, Sfax, Tunisia

## Abstract

**Introduction:**

Tobacco use remains a critical global public health challenge. Nursing students are of particular concern as they face high academic stress while being expected to serve as future role models for health promotion and mental health advocacy.

**Objectives:**

This study aimed to assess the prevalence of smoking among nursing students in Sfax, Tunisia, and to examine associations between nicotine dependence and health-promoting lifestyle behaviors.

**Methods:**

A cross-sectional study was conducted during the academic year 2024–2025 among all nursing students enrolled at the Higher Institute of Nursing Sciences of Sfax (N=266). Data were collected using a sociodemographic questionnaire, the Fagerström Test for Nicotine Dependence (FTND), and the Health-Promoting Lifestyle Profile II (HPLP-II).

**Results:**

The prevalence of smoking was 16.2% (n=43), with a marked male predominance (93%). Cigarettes were the most common form (81.4%), followed by electronic cigarettes (69.8%) and waterpipe use (48.8%). Nicotine dependence was low in 34.9% of smokers and moderate in 30.2%. Smoking was most prevalent among first-year students (51.2%) and was significantly associated with alcohol consumption (p = 0.05). The mean global HPLP-II score was 2.47 ± 0.40, with the highest subscale scores for interpersonal relations (2.72 ± 0.46) and spiritual growth (2.54 ± 0.48), and the lowest for physical activity (2.17 ± 0.63). Nicotine dependence showed significant negative correlations with nutrition (r = –0.205, p < 0.05), interpersonal relations (r = –0.296, p < 0.01), and stress management (r = –0.296, p < 0.01).

**Conclusions:**

Tobacco use is prevalent among Tunisian nursing students and negatively impacts several lifestyle dimensions. These findings underscore the importance of integrating tobacco prevention and cessation initiatives into nursing curricula while enhancing stress management and promoting healthy coping strategies among future healthcare professionals.

**Disclosure of Interest:**

None Declared

